# Illustrations of Surgical Anatomy, with Explanatory References; Founded on the Work of M. Blandin

**Published:** 1831

**Authors:** 


Illustrations of Surgical Anatomy, with Explanatory References; founded on the Work of M. Blandin. By John G. M. Burt, Surgeon to the City Dispensary, &c. &c. &c. Engraved under the Direction of the Editor, by Messrs. James and John Johnstone. Edinburgh, Maclachlan and Stewart, 1831.

Amidst the multitude of anatomical plates of all descriptions which are daily issuing from the press, the student is distracted, and unless his purse be longer than it usually is, he can purchase but a very small proportion of the whole. Some are of a higher character and more general utility than others, and some, from good fortune or superior merit, obtain a better name, and, what is more germain to the purpose, a wider circulation. Far be it from us to particularize; suffice it that such is the case. The present work consists of eight fasciculi, each containing two plates. They contain the surgical anatomy of the head and neck, the eye, the upper and lower extremities, and particularly of that chief domain of surgery, the pelvis. They are copper-plate, the drawings accurate, the plates themselves distinct and clear. Our own opinion respecting the value of anatomical plates is on record, and we need not repeat it here, but we may state that the present series are very good of their kind, and calculated, so far as plates can go, to prove of service to the operative surgeon. On these grounds we can recommend it, and to those who do not possess the work of M. Blandin, the present will prove acceptable.

